# Does chronic metformin therapy offer cardiomyoprotection in coronary artery bypass grafting surgery patients? A case-control study

**DOI:** 10.1186/cc12390

**Published:** 2013-03-19

**Authors:** A Delaporte, JP Hacquebard, P Origer, I Pastijn, M Maatouk, O Germay, E Stevens, P Kapessidou, A Roman

**Affiliations:** 1 CHU Saint-Pierre, Brussels, Belgium

## Introduction

Metformin, widely used as an antidiabetic drug, activates the AMP activated protein kinase, a key regulator of the metabolism providing protection under fuel deficiency. Chronic metformin therapy has been shown in long-term follow-up clinical studies to reduce cardiovascular mortality [1]. In animal experiments, acute metformin pretreatment has been shown to reduce ischemia-reperfusion injury on cardiomyocytes [2]. We want to evaluate whether outcomes are affected in coronary artery bypass grafting (CABG) surgery.

## Methods

We performed a retrospective case-control study on 341 patients admitted exclusively for CABG surgery with cardiopulmonary bypass between January 2006 and April 2012. Eighty-one patients chronically on metformin were matched 1:3 with nonmetformin-treated ones using gender, age, body mass index and number of arterial bypass grafts. Global mortality, ICU-free 28 postoperative days and creatinine kinase MB (CKMB) at postoperative baseline, 8 and 18 hours post baseline were compared.

## Results

Metformin-treated patient mortality was not significantly lower than in nonmetformin (1.2% vs. 3.1%, *P *= 0.24, Fischer exact test). ICU-free 28 postoperative days were higher in the metformin group (median 23.4 days; 95% CI = 22.5 to 24.3) than in the nonmetformin group (median 22.9 days; 95% CI 22.3 to 23.5, *P *0.04, Wilcoxon test). Median CKMB levels at postoperative baseline were 21 versus 26 ng/ml (*P *0.003), at 8 hours were 22 versus 53 ng/ml (*P *0.004) and 18 hours post baseline were 21.5 versus 27.5 ng/ml (*P *0.012). See Table 1.

**Table 1:** Case-control analysis

			**Statistics**,
	Metformin	Nonmetformin	*P *value
Mortality	1/81	8/260	0.24
28 days free ICU (days)	23.4	22.9	<0.04
CKMB T 0 hours (ng/ml)	21	26	<0.003
CKMB T+8 hours (ng/ml)	22	53	0.004
CKMB T+18 hours (ng/ml)	21.5	27.5	<0.012

## Conclusion

These data suggest that chronic preoperative treatment with metformin is associated with lower CKMB levels at postsurgery baseline, 8 and 18 hours post baseline with a shorter ICU stay in patients receiving CABG surgery under extracorporeal circulation.
